# Food Literacy as an Instrument for Health Promotion Among Hospital Workers: The “ABCibi Project”

**DOI:** 10.3390/nu17091515

**Published:** 2025-04-29

**Authors:** Samar El Sherbiny, Valeria Bellisario, Elena Lenta, Giacomo Scaioli, Giulia Squillacioti, Anna Rovera, Patrizia Lemma, Cloè Dalla Costa, Roberto Bono

**Affiliations:** 1 Department of Public Health and Pediatrics, University of Turin, Via Santena 5bis, 10126 Turin, Italy; samar.elsherbiny@unito.it (S.E.S.); giacomo.scaioli@unito.it (G.S.); giulia.squillacioti@unito.it (G.S.); patrizia.lemma@unito.it (P.L.); roberto.bono@unito.it (R.B.); 2 Clinical Nutrition Unit, Michele and Pietro Ferrero Hospital, 12060 Verduno, Italy; elenta@aslcn2.it (E.L.); cdallacosta@aslcn2.it (C.D.C.); 3 Research Center for Training, Health Education and Local Empowerment, University of Turin, 10126 Turin, Italy; 4 Fondazione Ospedale Alba-Bra Onlus, 12051 Alba, Italy

**Keywords:** food literacy, public health, health promotion, occupational health, health literacy

## Abstract

**Background**: Health literacy (HL) promotes the achievement of skills and information useful to endorse health. Food Literacy (FL) is a subtype of HL related to the knowledge necessary to achieve a healthy diet. **Methods**: This pilot study aimed to assess and improve FL of hospital workers through a survey before and after an educational intervention consisting of nutrition courses, infographics, and updates to the canteen service. FL was evaluated with a questionnaire, and Kruskal–Wallis, Friedman and Wilcoxon test was performed to assess group differences. **Results**: Of 897 participants, 375 (T1) completed both surveys, while 522 completed only T0. A pairwise comparison stratified by role, age and education revealed a significant improvement in FL scores in the T1 group. Improvements were observed in the T1 group, with overall scores rising from 6.2 ± 2.7 to 6.9 ± 2.1 (*p*-value < 0.001). An upgrade was noted in healthcare workers (T0 = 6.5 ± 2.2 vs. T1 = 7 ± 2, *p*-value < 0.001), administrative workers (T0 = 5.5 ± 2.5 vs. T1 = 6.2 ± 2.4, *p*-value = 0.008), all age groups (T0 = 6.3 ± 2.4 vs. T1 = 6.8 ± 2, *p*-value = 0.03, T0 = 6.2 ± 2.3 vs. T1 = 6.7 ± 2.2, *p*-value = 0.02, T0 = 6.2 ± 2.3 vs. T1 = 7 ± 2, *p*-value = 0.003), low and high education (T0 = 5.7 ± 2.3 vs. T1 = 6.6 ± 2.1, *p*-value < 0.001, T0 = 6.6 ± 2.3 vs. T1 = 7.1 ± 2 *p*-value = 0.03). **Conclusions**: Our survey highlights the efficacy of a multifactorial intervention in enhancing FL and proves the importance of food health promotion within workplaces.

## 1. Introduction

Health literacy (HL) is defined as the ability to gain useful skills and information for everyday life and to promote and maintain good health [1,2,3]. Hence, people should be able to find health-related information and apply this knowledge after a critical evaluation to stay healthy [2]. HL is an important component of public health [4] and is helpful in preventing the spread of many chronic communicable and non-communicable diseases, defined as conditions that last one year or more [5] like cardiovascular diseases, diabetes, and different types of cancer [2,5,6,7] which require medical attention and are often related to lifestyle behaviors that can be improved [5]. HL can be split into different areas, such as mental health, digital and media literacy, and functional healthcare knowledge [6]. Among several subtypes, this work focuses on food and nutrition literacy since diet, identified as one of the main risk factors for non-communicable diseases, together with smoking and physical inactivity, is the one that can be easily managed as part of lifestyle behaviors in a work environment [5,7]. Food Literacy (FL) refers to a knowledge set necessary for people to achieve and preserve a healthy diet [8]. Following Krause et al., 2016 [8], in this work, we will assume nutrition literacy as a subgroup of FL. FL is defined as the wealth of knowledge necessary to make conscious decisions to improve our dietary intake [6,9] and is composed of many elements: how to create a healthy eating plate and how to select quality products to make nourishing and balanced meals [10]. Several factors contribute to FL and food choices, extending beyond eating habits. Socioeconomic and cultural influences play pivotal roles in shaping dietary behaviors [11,12]; in fact, strong correlations between FL levels and education have been demonstrated [13,14]. Additionally, different age groups make different food choices influenced by health concerns or factors such as convenience and affordability [15]. A healthy and balanced diet is a cost-effective disease prevention method that is also helpful for avoiding pharmaceutical intervention [16]. An example of the beneficial effects of a correct and balanced diet is demonstrated by the adoption of a Mediterranean dietary pattern, as demonstrated by trials like the PREDIMED [17] or the Moli-sani cohort [18]. The goal of FL should be to improve overall health by reducing food-related diseases [9]. This aim should be included in different contexts, such as in the framework of workplace health promotion initiatives. Food consumption is a daily life activity [10], and for most workers, especially in a healthcare setting like one of the “ABCibi” projects, at least one of the daily meals is consumed at work, often provided by the company through a canteen service. Therefore, a canteen that provides workers with healthy food choices is crucial. The aforementioned concepts have been considered in our study to better understand employees’ educational needs. The aim is to investigate the FL of hospital workers before and after intervention at multiple levels: administration of nutrition education courses, infographics, and a website, coupled with the renewal of the food offered at the workplace as a direct intervention to offer healthier meals. After that, a new survey was conducted using the same modalities to verify whether and how the participants improved their FL knowledge. This survey was significant for the hospital administration in preserving the health and wellness of their employees and having workers well-trained about food topics, especially those who deal with patient care every day.

## 2. Materials and Methods

The “ABCibi” project is a repeated and paired cross-sectional study involving 897 workers. The first survey was administered in 2021, while the second was proposed to workers in 2022 after multiple interventions. The main changes were educational courses aimed at enhancing employees’ basic FL, which are crucial for making informed and healthy dietary choices; infographics to promote FL within the hospital; canteen service renewal; and a dedicated website. The last three were permanent changes in the hospital, while the educational courses took place during the year between the two surveys, delivered by professional dietitians.

### 2.1. Population and Recruitment

The survey was conducted at the “Michele and Pietro Ferrero” Hospital in Piedmont (Italy). Recruitment was performed using a link shared with the workers from the hospital administration and by placing QR codes inside the hospital to reach as many workers as possible. Participation was entirely anonymous, no identification codes were assigned to respondents, and the survey was automatically closed if the workers were not A.S.L. employees. Informed consent was obtained from all participants, who expressed their agreement by scanning the QR code and completing the survey, thereby consenting to their participation and the use of the information provided. Considering that the survey was exclusively restricted to hospital employees, the Head of the Joint Ethics Committee of Alessandria Hospitals has confirmed that no ethical approval was required (following the 2020–2023 regulation of the Ethics Committee). The Data Protection Office (DPO) and the Hospital Administration reviewed and approved the survey, also concluding that ethical approval was unnecessary because no personal data was involved. The collected data were used in an aggregated and anonymous form in compliance with the Personal Data Protection Code (Legislative Decree No. 196/2003), which was updated with the new Legislative Decree No. 101/2018 to adapt the Italian legislation to the European Privacy Regulation (EU Regulation No. 679/2016, GDPR).

### 2.2. Data Collection and Analysis

Few data sets concerning FL are available in Italy; in 2021, a FL questionnaire was validated in Italy [16], but the tool was unavailable when this study began. Therefore, a questionnaire was created to investigate the workers’ knowledge of nutrients and the correct composition of the ideal plate [19]. The web interface REDCap (Research Electronic Data Capture) was used to create the survey and collect the data. The questionnaire was sub-grouped into three sections: demographic information, FL and customer satisfaction with the new food service. General information (age, sex, education, working area, and nationality) about the employees who participated in the survey was collected in agreement with DPO dispositions; hence, only the age category could be collected. A second section collected information on satisfaction perceived by the workers who attended the canteen service before and after the renewal. The last part was dedicated to testing and scoring workers’ FL. FL was determined through six questions about macronutrients (two items), the fiber content of common foods (one item), the composition of the healthy eating plate (two items), and dairy consumption (one item). The total FL score of each subject was calculated as follows: four out of six questions were multiple choice, with only one correct answer; therefore, the score was 1 point if the answer was correct and 0 points if the answer was incorrect. The last two questions were composed of five sub-questions; hence, the answer was considered correct (1 point), with at least four correct answers. Scores out of six were converted to a decimal scale. The final score was used to perform the statistical analysis. To evaluate the effectiveness of our intervention, a pairing was performed by exclusively selecting participants who attended the second time point of the survey (T1 group) and who declared to have attended the first survey (T0 group). The demographic categories of the T1 survey provided us with enough information to select demographically similar subjects from the T0 group without violating the privacy agreement with the DPO. Hence, a third group of people, the T0–T1 group, who presumably answered both forms, was created and analyzed. The stratification criteria were obtained from the original survey to get standardized categories: Working roles were defined as (1) workers in the healthcare area and (2) workers in the administrative area and other working areas. The study participants were divided also into low, medium, and high educational levels and into three age groups: <30, 31–50, and >50 years old.

### 2.3. Statistical Analysis

The analysis of the data was carried out using STATA 16.1 software (StataCorp LLC: College Station, TX, USA) and GraphPad 8 software (GraphPad Boston, MA, USA). The demographic and personal characteristics of the subjects were summarized as absolute and relative frequencies for categorical variables and as Mean ± Standard Deviation (±SD) for continuous variables. Continuous variables are presented as Mean (±SD), while categorical variables are presented as frequencies, using both percentages and the number of observations. As the statistical distribution of the parameters was found to be non-Gaussian (Kolmogorov–Smirnov test), non-parametric tests were used to assess group differences (Kruskal–Wallis and Friedman tests) and paired-group comparison analyses (Wilcoxon tests). A two-sided *p*-value < 0.05 was considered to indicate statistical significance.

## 3. Results

### 3.1. General Population Description

Table 1 describes the general characteristics of the study population. A total of 897 subjects were enrolled, of which 375 subjects, hereafter called T0–T1 subjects, completed the two stages of the survey, while 522 subjects, hereafter called T0 subjects, completed only the initial stage. The sample was homogenous in terms of sex, age distribution and educational level, both for groups, with approximately 25% being male and almost 50% having a high-level education (i.e., graduation degree). 

#### 3.1.1. T0 Group Description

Overall, 522 hospital workers exclusively answered the first survey call. Table 1-Part A reports the main features of this study population. The distribution of the population was homogeneous except for gender (25.3% of males and 74.7% of females), but the stratification for sex in the subsequent results was not statistically significant. The most represented age range was between 31 and 50 y.o. (38.7% of the population). A total of 394 workers belonged to role 1, 128 to role 2, and more than half of the population belonged to a higher education group (57.7%), while 10.1% belonged to a low education group and 32.2% to a medium education group.

#### 3.1.2. T0-T1 Group Description

Of the 792 people who took part in the second survey, only 413 declared they had also completed the first survey in 2021. After the database cleaning, 375 workers reported having completed both survey calls without any missing information, 73.6% of whom were female (Table 1-Part B); hence, they were included in this work to perform the pairing with the first survey. Among them, the majority (38.7%) of the individuals were aged 31–50 years. Role 1 was the most represented, with 298 workers, whereas 77 workers belonged to role 2. A higher education level represented 61.4% of the population, an intermediate education level represented 25.8%, and a lower education level represented 12.8%.

### 3.2. Food Literacy Score

Table 2 reports the cumulative FL subgrouped by the independent variables declared: stage, working role, sex, age and educational level. The mean score of the T0 group was 6.2/10 (SD ± 2.3). Overall, for the T0-T1 group, the FL score at T0 was 6.3/10 (SD ± 2.3), while that at T1 was 6.9/10 (SD ± 2.1), highlighting a significant difference between the two time points (Table 2-Part A; *p* < 0.005). Afterward, the mean FL score of the study groups was calculated considering the stratification criteria. In the T0 group, a significant difference between roles (Table 2-Part A; *p*-value = 0.003) and educational level (*p* value = 0.004) was highlighted. For the T0–T1 group, a significant difference was found within role 2 (Table 2-Part B; *p*-value = 0.02) and for low and high educational levels (Table 2-Part D; *p*-value = 0.04 and *p*-value = 0.03, respectively). There were no significant differences in age between the two groups.

### 3.3. Pairwise Comparison of FL Scoring

**Figure 1:**
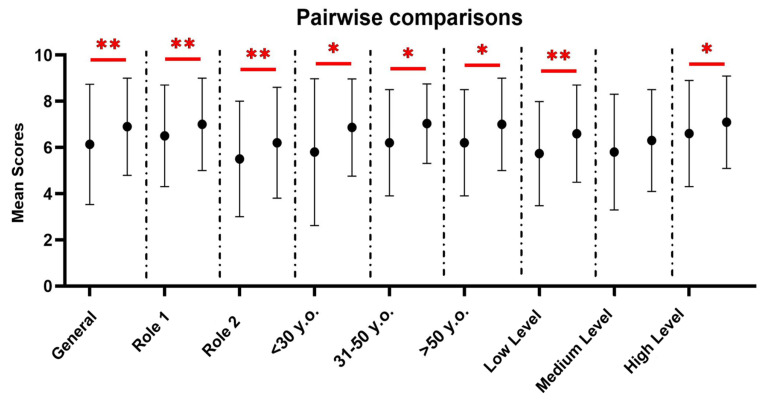
Pairwise comparisons (means ± S.D.s) of the general and stratified scores (working roles, age and educational levels). * *p* < 0.05, ** *p* < 0.01.

Table 3 and Figure 1 report the pairwise comparisons stratified by role, age and education. To identify potentially significant differences in the FL scores, the T0 group was compared with both the T0 and T1 groups of the T0–T1 group. The comparison between the T0 and T1 scores yielded a significant result for almost all categories except for medium education (*p*-value = 0.05). Overall, the findings highlighted a significant improvement in the T1 FL scores compared to those in the T0 group in the general population comparison (Table 3-Part A; *p*-value < 0.001). The same important result is evident by observing role 1 and role 2 (Table 3-Part A; *p*-value < 0.001 and *p*-value = 0.008, respectively), all age subgroups <30 y.o., 31–50 y.o. and >50 y.o. (Table 3-Part B; *p*-value = 0.03, *p*-value = 0.02, and *p*-value = 0.003, respectively), and, low and high education (Table 3-Part C; *p*-value < 0.001, and *p*-value = 0.03, respectively).

## 4. Discussion

The present study was designed and carried out at “Michele and Pietro Ferrero” Hospital between 2021 and 2022 to verify the effectiveness of a multifactorial intervention. This study sought to assess the effectiveness of a multifactorial intervention implemented between the two designated periods. The role, age group and education of the workers were evaluated. The baseline FL scores were homogeneous among the population at the T0 time point; indeed, the statistical tests gave no significant results in the pairwise comparison. Conversely, between people who completed only the T0 survey and people who completed both surveys, a significant improvement was found, highlighting the effectiveness of the intervention. Even in the observational nature of our study, we observed that the most influenced categories were also the targets from which we expected a significant result, i.e., older age groups and lower education levels. The intervention was particularly powerful and effective for employees older than 50 y.o., with low education in both healthcare and administrative working roles. These results are in line with previous research in which the influence of socioeconomic factors has been highlighted. The socioeconomic aspect, together with the educational level, are fundamental in determining food choices since, in most cases, food that is less healthy and nourishing is very cheap, hence more affordable for people with a lower financial availability than in most of the cases match with lower education [11,20]. The importance of socioeconomic determinants was underlined in the validation of a food choice value measurement by Lyerly and Reeve [11]. They tested the effect of individual factors on food decisions and how they impact health. Connors and colleagues [20] emphasized the presence of different key factors in food choice, and among them, we found that cost and convenience are related to different age groups. Some studies have shown that age significantly influences food choices [12,21]. Older people are more likely to choose healthier foods due to the positive impacts on their health, while young people are more prone to evaluate convenience in terms of cost-effectiveness and simplicity, neglecting qualitative aspects [12,15]. Thus, this can suggest why, in this study, the improvement in the score was greater for people older than 50 years. Another important factor is educational level. It can influence not only FL but also dietary intake and the onset of diet-related non-communicable diseases such as obesity, diabetes, and cardiovascular diseases [22,23,24], and our results are in line with this assumption. The introduction of new, unfamiliar foods and awareness about the existence of healthier alternatives to some ultra-processed products strictly depend on educational level and income [15]. Additionally, convenience plays an important role in food choice, especially in populations with low education, since education is a determinant of future employment and income [12,25]. Our results emphasize that the intervention’s most significant impact is observed among individuals with lower education levels. Finally, we observe a significant enhancement across both working role categories, likely resulting from the combined influence of age groups and education levels.

The survey’s scores tell us that, while we encountered an improvement, further efforts are needed, particularly regarding the composition of the healthy meal, where no improvement has been noted. According to the FL definition of Vidgen and Gallegos [10], the ability to combine foods to create a healthy, nourishing meal is one of the main skills that should be provided by FL. Hence, the issue of meal composition should be further stressed. Food choices are directly related to eating behavior, and by improving the knowledge of food composition and meal preparation, some detrimental habits can be counteracted, leading to a general improvement in people’s health and well-being [12]. These findings seem to be relevant not only from a literacy point of view but also for improving our knowledge about the identification of a target population that is in need of further health and food education. Thus, promoting FL helps prevent diseases and promotes health.

### Strengths and Limitations

This study’s strength is the large number of participants. This is the first hospital in Piedmont in which the food offered to workers was improved to be sustainable and promote food culture as an instrument for prevention. The main limitation is the lack of individual linkage between the two surveys due to privacy restrictions and the need to ensure the full protection of employees’ anonymity and confidentiality, key principles in the context of internal surveillance and organizational well-being assessment. Although we attempted to align responses from the T0 and T1 surveys based on key demographic variables, individual data pairing could not be performed due to strict privacy and data protection regulations imposed by the hospital administration, which did not allow the use of identifiable or traceable information, even in encrypted form. Thus, it was impossible to verify the effectiveness of the intervention for single workers, but we were able to consider the results of the groups. Despite this limitation, we believe the study still provides useful preliminary insights that can inform future, more rigorously designed longitudinal evaluations.

Secondly, the employed questionnaire was not validated at the time of data collection, as it was specifically designed and tailored to address the immediate and context-specific needs of the hospital’s internal monitoring initiative. In fact, the primary aim of this initiative was not research-oriented but rather intended as an institutional tool to systematically assess and monitor the well-being and perceptions of hospital personnel, with the ultimate goal of informing management strategies and improving working conditions and service delivery during a particularly challenging period.

## 5. Conclusions

This interventional study aimed at studying and, at least, trying to prevent poor nutritional habits in hospital workers and highlighted that a multifactorial intervention is effective in improving FL. The high participation rate demonstrated the interest of the workers in these themes. Public health and health promotion at the occupational level often neglect the impact that some aspects of employees’ lifestyles, such as diet, can have on their work lives. Furthermore, the environmental impact of making conscious food choices is crucial, as it can aid in preventing food waste and supporting workers in making more sustainable decisions regarding food in their everyday lives. This is important in a hospital setting, where food waste is prevalent due to the large number of staff and patients served by the canteen. In conclusion, we must initiate additional projects aimed at enhancing the community’s understanding of these subjects, particularly within workplaces dedicated to disease treatment and health promotion.

## Figures and Tables

**Table 1:** General description of the study population sub-grouped as part A/T0 subjects and part B/T0ߝT1 subjects; n = sample size, y.o. = years old.

		Sex		Age		Educational Level	
Part A: T0 subjects (n = 522)		Male	132 (25.3%)	<30 y.o.	71 (14.6%)	Low level	53 (10.1%)
				31–50 y.o.	202 (38.7%)	Medium level	168 (32.2%)
		Female	390 (74.7%)				
				>50 y.o.	159 (30.5%)	High level	301 (57.7%)
	Role 1 (n = 394)	Male	94 (23.8%)	<30 y.o.	(14.6%)	Low level	35 (8.9%)
				31–50 y.o.	202 (38.7%)	Medium level	94 (23.8%)
		Female	300 (76.2%)				
				>50 y.o.	159 (30.5%)	High level	265 (67.1%)
	Role 2 (n = 128)	Male	39 (23%)	<30 y.o.	71 (14.6%)	Low level	17 (11%)
				31–50 y.o.	202 (38.7%)	Medium level	74 (61%)
		Female	89(77%)				
				>50 y.o.	159 (30.5%)	High level	37 (28%)
Part B: T0-T1 subjects (n = 375)		Male	99 (26.4%)	<30 y.o.	71 (14.6%)	Low level	40 (12.8%)
				31–50 y.o.	202 (38.7%)	Medium level	81 (25.8%)
		Female	276 (73.6%)				
				>50 y.o.	159 (30.5%)	High level	193 (61.4%)
	Role 1 (n = 298)	Male	63 (21.2%)	<30 y.o.	90 (14.6%)	Low level	44 (14.7%)
				31–50 y.o.	79 (38.7%)	Medium level	51 (17.2%)
		Female	235 (78.8%)				
				>50 y.o.	129 (30.5%	High level	203 (68.1%)
	Role 2 (n = 77)	Male	36 (46.8%)	<30 y.o.	17 (14.6%)	Low level	3 (3.6%)
				31–50 y.o.	26 (38.7%)	Medium level	44 (57.1%)
		Female	41 (53.2%)				
				>50 y.o.	34 (30.5%)	High level	30 (39.3%)

**Table 2:** The cumulative food literacy scores of the two study groups were subgrouped by the independent variables: role, sex, age and education.

Food Frequency Total Score				
Part A: working roles				
		Total score (Mean ± SD)		Statistical analyses (Kruskal–Wallis test)
T0 subjects		6.2 ± 2.3		
	Role 1	6.5 ± 2.2		*p* = 0.003
	Role 2	5.5 ± 2.5		
T0–T1 subjects		T0	T1	Statistical analyses (Friedman test)
		6.3 ± 2.3	6.9 ± 2.1	2.8 *p* < 0.005
	Role 1	6.6 ± 2.2	7 ± 2	1.9 *p* = 0.05
	Role 2	5.4 ± 2.4	6.2 ± 2.4	2.9 *p* = 0.02
Part B: sex				
		Total score (Mean ± SD)		Statistical analyses (Kruskal–Wallis test)
T0 subjects				
	Male	5.5 ± 2.6		*p* = 0.056
	Female	6.5 ± 2.2		
T0-T1 subjects				Statistical analyses (Friedman test)
	Male	6 ± 2.5	6.4 ± 2.4	0.5 *p* = 0.4
	Female	6.5 ± 2.2	7 ± 2	3.7 *p* = 0.05
Part C: age				
		Total score (Mean ± SD)		Statistical analyses(Kruskal–Wallis test)
T0 subjects				
	<30 y.o.	6.3 ± 2.4		*p* = 0.5
	31–50 y.o.	6.2 ± 2.3		
	>50 y.o.	6.2 ± 2.3		
T0-T1 subjects		T0	T1	Statistical analyses (Friedman test)
	<30 y.o.	6.3 ± 2.3	6.8 ±2	0.3 *p* = 0.5
	31–50 y.o.	6.3 ± 2.3	6.7 ± 2.2	2.2 *p* = 0.1
	>50 y.o.	6.5 ± 2.3	7 ± 2	1.6 *p* = 0.1
Part D: educational level				
		Total score (Mean ± SD)		Statistical analyses(Kruskal–Wallis test)
T0 subjects				
	Low level	5.7 ± 2.3		*p* = 0.004
	Medium level	5.8 ± 2.5		
	High level	6.6 ± 2.3		
T0–T1 subjects		T0	T1	Statistical analyses(Friedman test)
		6.7 ± 2.3	6.7 ± 2.1	2.03 *p* = 0.4
	Low level	5.6 ± 2.4	6.6 ± 2.1	2.9 *p* = 0.04
	Medium level	6 ± 2.4	6.3 ± 2.2	0.3 *p* = 0.6
	High level	6.7 ± 2.2	7.1 ± 2	2.25 *p* = 0.03

**Table 3:** A pairwise comparison between T0 and T1 subjects was stratified by role, age, and educational level.

Pairwise Comparison T0 Subjects vs. T0–T1 Subjects				
				Statistical Analyses (Wilcoxon Test)
	T0 only vs. T0	6.2 ± 2.7	6.3 ± 2.3	*p* = 0.22
	T0 only vs. T1	6.2 ± 2.7	6.9 ± 2.1	*p* < 0.001
Part A: Stratification per ROLE				
ROLE 1				
	T0 only vs. T0	6.5 ± 2.2	6.6 ± 2.2	*p* = 0.4
	T0 only vs. T1	6.5 ± 2.2	7 ± 2	*p* < 0.001
ROLE 2				
	T0 only vs. T0	5.5 ± 2.5	5.4 ± 2.4	*p* = 0.2
	T0 only vs. T1	5.5 ± 2.5	6.2 ± 2.4	*p* = 0.008
Part B: Stratification per AGE CLASSES				
<30 y.o.				
	T0 only vs. T0	6.3 ± 2.4	6.3 ± 2.3	*p* = 0.4
	T0 only vs. T1	6.3 ± 2.4	6.8 ± 2	*p* = 0.03
31–50 y.o.				
	T0 only vs. T0	6.2 ± 2.3	6.3 ± 2.3	*p* = 0.6
	T0 only vs. T1	6.2 ± 2.3	6.7 ± 2.2	*p* = 0.02
>50 y.o.				
	T0 only vs. T0	6.2 ± 2.3	6.5 ± 2.3	*p* = 0.2
	T0 only vs. T1	6.2 ± 2.3	7 ± 2	*p* = 0.003
Part C: Stratification per EDUCATIONAL LEVELS				
LOW LEVEL				
	T0 only vs. T0	5.7 ± 2.3	5.6 ± 2.4	*p* = 0.3
	T0 only vs. T1	5.7 ± 2.3	6.6 ± 2.1	*p* < 0.001
MEDIUM LEVEL				
	T0 only vs. T0	5.8 ± 2.5	6 ± 2.4	*p* = 0.7
	T0 only vs. T1	5.8 ± 2.5	6.3 ± 2.2	*p* = 0.05
HIGH LEVEL				
	T0 only vs. T0	6.6 ± 2.3	6.7 ± 2.2	*p* = 0.2
	T0 only vs. T1	6.6 ± 2.3	7.1 ± 2	*p* = 0.03

## Data Availability

The datasets generated and analyzed during the current study are not publicly available due to the privacy policy determined by the hospital DPO.

## Abbreviations

The following abbreviations are used in this manuscript:

HL	Health Literacy
FL	Food Literacy
DPO	Data Protection Office
SD	Standard Deviation
